# Genotypic diversity among multidrug resistant *Pseudomonas aeruginosa* and *Acinetobacter* species at Mulago Hospital in Kampala, Uganda

**DOI:** 10.1186/s13104-017-2612-y

**Published:** 2017-07-14

**Authors:** David P. Kateete, Ritah Nakanjako, Moses Okee, Moses L. Joloba, Christine F. Najjuka

**Affiliations:** 1 Department of Immunology and Molecular Biology, College of Health Sciences, Makerere University, Kampala, Uganda; 2 Department of Medical Microbiology, College of Health Sciences, Makerere University, Kampala, Uganda

**Keywords:** Sub-Saharan Africa, Hospital environment, Hospitalized patients, Gram negative bacteria, Antimicrobial resistance AMR, Carbapenems, Genotyping, Rep-PCR

## Abstract

**Background:**

Multidrug resistant *Pseudomonas aeruginosa* and Acinetobacter species are common causes of nosocomial infections worldwide. Recently we reported the occurrence of carbapenem resistant Enterobacteriaceae, *P. aeruginosa* and *Acinetobacter* species at Mulago National Referral Hospital in Kampala, Uganda, but the isolates were not analyzed for genetic relatedness. Herein we report the intra-species genotypic diversity among *P. aeruginosa* and *Acinetobacter baumannii* isolated from hospitalized patients and the environment at Mulago Hospital, using repetitive elements-based PCR (Rep-PCR) genotyping.

**Results:**

A total of 736 specimens from hospitalized patients were processed for culture and sensitivity testing yielding 9 (1.2%) *P. aeruginosa* and 7 (0.95%) *A. baumannii*. Similarly, 100 samples from the hospital environment were processed yielding 33 (33%) *P. aeruginosa* and 13 (13%) *A. baumannii*. Altogether, 62 non-repetitive isolates were studied (42 *P. aeruginosa* and 20 *A. baumannii*), of which 38% (16/42) *P. aeruginosa* and 40% (8/20) *A. baumannii* were multidrug resistant (isolates resistant to three or more classes of antimicrobials). Carbapenem resistance prevalence was 33 and 21% for *P. aeruginosa* from patients and the environment, respectively, while it was 14 and 86% for *A. baumannii* from patients and environment, respectively. Cluster analysis of the Rep-PCR fingerprints revealed a high level of genetic diversity among the isolates within each species as few isolates were clustered (at 100% level of similarity). More to this, the clustered isolates revealed a complex nature of multidrug resistant *P. aeruginosa* and *A. baumannii* clones circulating at Mulago Hospital. Importantly, certain isolates from the environment and patients were clustered, implying that hospitalized patients at Mulago were probably infected with strains from the environment.

**Conclusions:**

The prevalence of multidrug resistant *P. aeruginosa* and *A. baumannii* is high at Mulago Hospital but carbapenem resistance prevalence remains relatively low in isolates from hospitalized patients. Importantly, the prevalence of carbapenem resistance in isolates from the environment is high implying the infection control practices at the hospital might be inadequate.

**Electronic supplementary material:**

The online version of this article (doi:10.1186/s13104-017-2612-y) contains supplementary material, which is available to authorized users.

## Background

*Pseudomonas aeruginosa* and *Acinetobacter* species are ubiquitous in hospital environments worldwide, and they are frequently implicated among the commonest causes of nosocomial infections that are difficult to treat. This is attributed to the fact that these organisms possess inherent drug resistance mechanisms (e.g. constitutive expression of AmpC beta-lactamases and efflux pumps, low permeability of the outer membrane, etc.), and they are also capable of acquiring additional resistance mechanisms to multiple classes of antimicrobials (e.g. beta-lactams, aminoglycosides and fluoroquinolones) through horizontal gene transfer [1–3]. Carbapenems have been the most effective drugs used to treat infections caused by multidrug resistant Gram negative bacteria including *P. aeruginosa* and Acinetobacter species. However, the emergence of carbapenemase producing strains, majority of which are multidrug resistant, is threatening the use of carbapenems in the management of such infections [4, 5].

Carbapenemase producing *P. aeruginosa* and Acinetobacter were first described in the 1990s and many carbapenemase producing Gram-negative species are increasingly being reported worldwide with increasing rates [4–7]. Of concern, carbapenemase producing Gram negative species appear to be rapidly spreading in sub-Saharan Africa [5, 8–11] pointing to the need of regular surveillance for carbapenem resistant bacteria in hospitals and/or healthcare facilities in low-income settings. Recently, we reported the occurrence of carbapenem resistant Enterobacteriaceae, *P. aeruginosa* and *Acinetobacter baumannii* at Mulago National Referral Hospital in Kampala, Uganda [8, 10]. However, the isolates were not analyzed for genetic relatedness. Herein we report the intra-species genotypic diversity among *P. aeruginosa* and *A. baumannii* isolated from hospitalized patients and the environment at Mulago Hospital, using repetitive elements-based PCR (Rep-PCR) genotyping. Cluster analysis revealed a high level of genetic diversity as few isolates were clustered at ≥85% level of similarity. More to this, the few clustered isolates revealed a complex nature of multidrug resistant *P. aeruginosa* and *A. baumannii* clones circulating at Mulago Hospital.

## Results

Between September 2012 and October 2013, a total of 736 specimens from hospitalized patients were processed for culture and sensitivity testing yielding 9 (1.2%) *P. aeruginosa* and 7 (0.95%) *A. baumannii* (all non-repetitive isolates). *P. aeruginosa* was isolated from tracheal aspirates (3), urine (2), pus (2), ear swab (1) and sputum (1), while *A. baumannii* was isolated from sputum (3), ear swabs (2), and tracheal aspirates (2). On the other hand, 100 samples from the hospital environment (surgical/medical wards including the intensive care units, ICUs) that were processed yielded 33 (33%) *P. aeruginosa* and 13 (13%) *A. baumannii*. Altogether, 62 non-repetitive isolates were characterized (42 *P. aeruginosa* and 20 *A. baumannii*).

### Antimicrobial susceptibility profiles

The antimicrobial susceptibility patterns among the isolates are shown in Tables 1 and 2, and Additional file 1: Table S1. None of the isolates from patients were pan-susceptible. The overall prevalence of resistance was: amikacin 14%, imipenem (24%), gentamicin 43%, ceftazidime 45%, cefepime 7%, aztreonam 48%, piperacillin/tazobactam 62%, ciprofloxacin 48% for *P. aeruginosa*; amikacin 15%, gentamicin 35%, meropenem 5%, ceftazidime 30%, cefepime 5%, aztreonam 40%, piperacillin/tazobactam 70%, ciprofloxacin 40%, and trimethoprim/sulfamethoxazole (SXT) 60% for *A. baumannii*. All *P. aeruginosa* isolates were susceptible to meropenem while three isolates from patients and seven from the environment were imipenem resistant, Table 1.

**Table 1:** Antimicrobial resistance rates among *P. aeruginosa* (% resistant)

Antimicrobial	Hospitalized patients n = 9	Environment n = 33	Total N = 42
Beta-lactam agents			
Aztreonam	4 (44%)	16 (48%)	20 (48%)
Cefepime	3 (33%)	0	3 (7%)
Ceftazidime	5 (56%)	14 (42%)	19 (45%)
Imipenem	3 (33%)	7 (21%)	10 (24%)
Meropenem	0	0	0
Piperacillin–tazobactam	5 (56%)	21 (64%)	26 (62%)
Aminoglycosides			
Amikacin	2 (22%)	4 (12%)	6 (14%)
Gentamicin	5 (56%)	13 (39%)	18 (43%)
Quinolones			
Ciprofloxacin	7 (78%)	13 (39%)	20 (48%)

**Table 2:** Antimicrobial resistance rates among *A. baumannii* (% resistant)

Antimicrobial	Hospitalized patients n = 7	Environment n = 13	Total N = 20
Beta-lactam agents			
Aztreonam	3 (43%)	5 (34%)	8 (40%)
Cefepime	1 (14%)	0	1 (5%)
Ceftazidime	5 (71%)	1 (8%)	6 (30%)
Imipenem	1 (14%)	6 (86%)	7 (35%)
Meropenem	1 (14%)	0	1 (5%)
Piperacillin/tazobactam	3 (43%)	11 (85%)	14 (70%)
Aminoglycosides			
Amikacin	3 (43%)	0	3 (15%)
Gentamicin	5 (71%)	2 (15%)	7 (35%)
Quinolones			
Ciprofloxacin	6 (86%)	2 (15%)	8 (40%)
Others			
Trimethoprim/sulfamethoxazole (SXT)	4 (57%)	8 (62%)	12 (60%)

Overall, carbapenem resistance prevalence was 33 and 21% for *P. aeruginosa* from patients and the environment, respectively, while it was 14 and 86% for *A. baumannii* from patients and environment, respectively. Furthermore, all the imipenem resistant *P. aeruginosa* isolates were also resistant to aztreonam while 90% were resistant to piperacillin/tazobactam. On the other hand, all carbapenem resistant *A. baumannii* were resistant to piperacillin/tazobactam while 57% were aztreonam resistant. Of note, SXT-susceptible, carbapenem resistant *A. baumannii* from patients and the environment was noted, Table 2 and Additional file 1: Tables S1, Additional file 2: Table S2. Furthermore, 38% (16/42) of *P. aeruginosa* and 40% (8/20) of *A. baumannii* isolates were multidrug resistant (resistance to three or more antimicrobial classes [12]).

### Beta-lactamases

Overall, 67% of the carbapenem resistant isolates were positive with the imipenem/EDTA double disk synergy test, implying the isolates were metallo-beta-lactamase producers, Additional file 1: Table S1. Hence, metallo-beta-lactamases were responsible for carbapenem resistance in majority of the carbapenem resistant isolates studied. Furthermore, 60% (37/62) of the isolates were resistant either to aztreonam or ceftazidime (extended spectrum cephalosporins), implying they were suspects for AmpC (class C β-lactamase enzyme/cephalosporinase) and extended spectrum beta-lactamases (ESBLs) production. However, none of these isolates was positive on ESBL and AmpC screening.

### Genotypic diversity

Genotypic diversity within each species was investigated using Rep-PCR genotyping. Rep-PCRs produced 0–15 consistent polymorphic bands per isolate of sizes 250–9400 bp (BOXAIR-PCR), 90–8000 bp (ERIC-PCR), and 100–6557 bp (REP-PCR), Additional file 3: Figure S1.

**Fig. 1:**
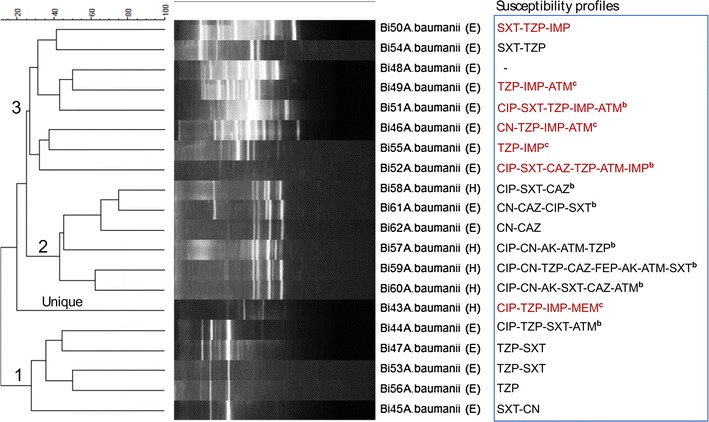
Dendrogram based on Dice’s coefficient of similarity using UPGMA method implemented by the Bionumerix program showing relationships between *A. baumannii* isolates according to BOXAIR-PCR genotyping. *E* isolate from hospital environment; *H* isolate from hospitalized patient. The susceptibility profiles (extreme *right panel*) for carbapenem-resistant isolates are indicated in *brown font*. *AK* amikacin, *CN* gentamicin, *IMP* imipenem, *MEM* meropenem, *CAZ* ceftazidime, *FEP* cefepime, *ATM* aztreonam, *TZP* piperacillin/tazobactam, *CIP* ciprofloxacin, *SXT* trimethoprim/sulfamethoxazole. ^b^Multidrug-resistance pattern; ^c^SXT-susceptible carbapenem-resistant *A. baumannii*

**Fig. 2:**
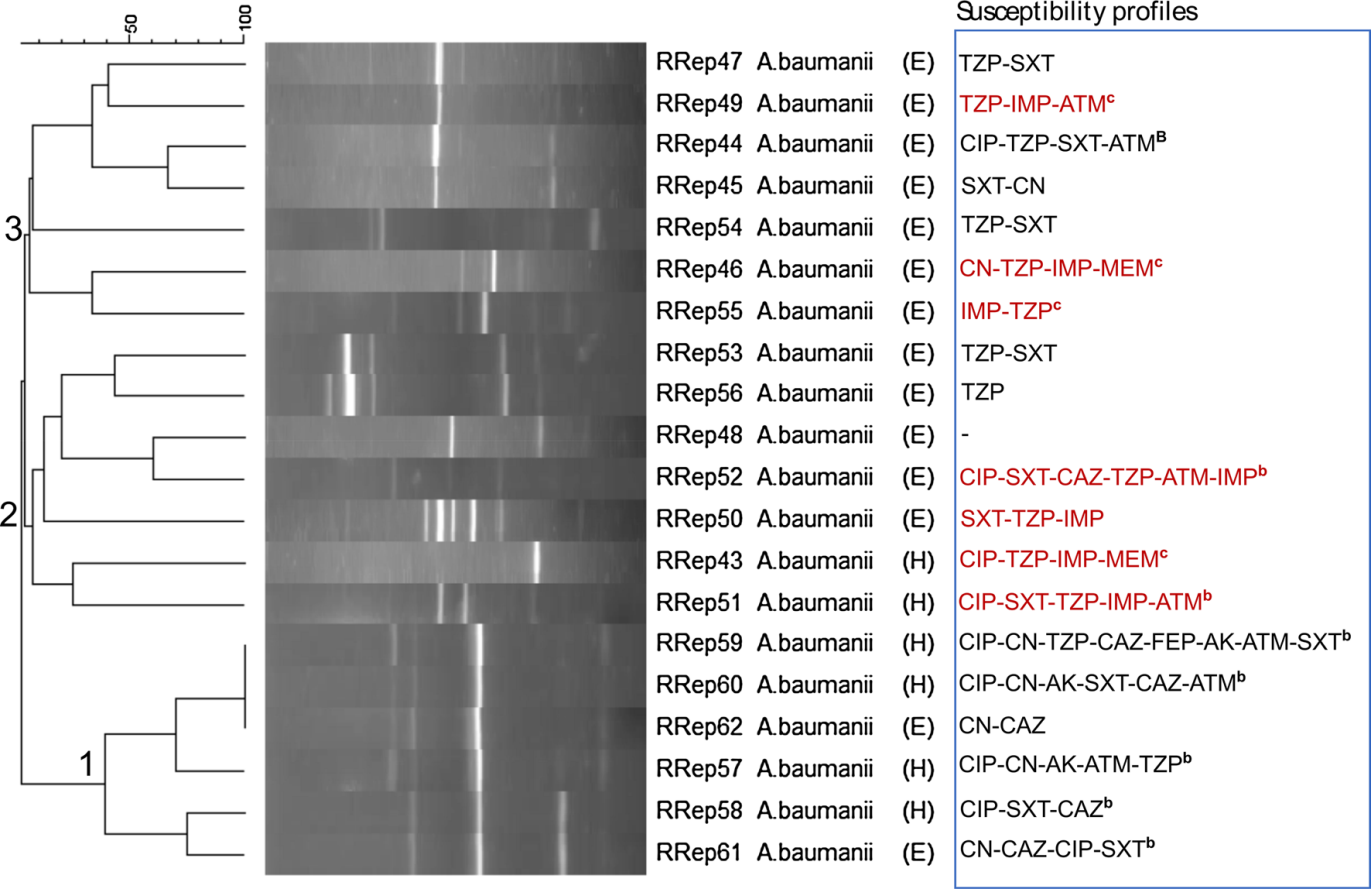
Dendrogram based on Dice’s coefficient of similarity using UPGMA method implemented by the Bionumerix program showing relationships between *A. baumannii* isolates according to REP-PCR genotyping. *E* isolate from hospital environment; *H* isolate from hospitalized patient. The susceptibility profiles (extreme *right panel*) for carbapenem-resistant isolates are indicated in *brown font*. *AK* amikacin, *CN* gentamicin, *IMP* imipenem, *MEM* meropenem, *CAZ* ceftazidime, *FEP* cefepime, *ATM* aztreonam, *TZP* piperacillin/tazobactam, *CIP* ciprofloxacin, *SXT* trimethoprim/sulfamethoxazole. ^b^Multidrug-resistance pattern; ^c^SXT-susceptible carbapenem-resistant *A. baumannii*

At 100% similarity scale, BOXAIR-PCR fingerprints for *A. baumannii* isolates were not clustered (Fig. 1); more to this for Acinetobacter, REP-PCR fingerprints produced only one cluster (Fig. 2) while ERIC-PCR fingerprints produced two clusters (Additional file 4: Figure S2). Isolates 59 and 62 (both carbapenem susceptible) from a patient and environment, respectively, were considered genetically related in that their fingerprints were clustered at 100% similarity scale by both ERIC-PCR and REP-PCR cluster analysis, Fig. 2 and Additional file 4: Figure S2. Altogether, only five Acinetobacter isolates were clustered at 100% similarity scale. This implies low level of genetic relatedness among the isolates. More to this, the few clustered isolates also reveal a complex nature of *A. baumannii* multidrug resistant clones circulating at Mulago Hospital in that the isolates exhibited diverse antimicrobial susceptibility phenotypes, Additional file 2: Table S2.

**Fig. 3:**
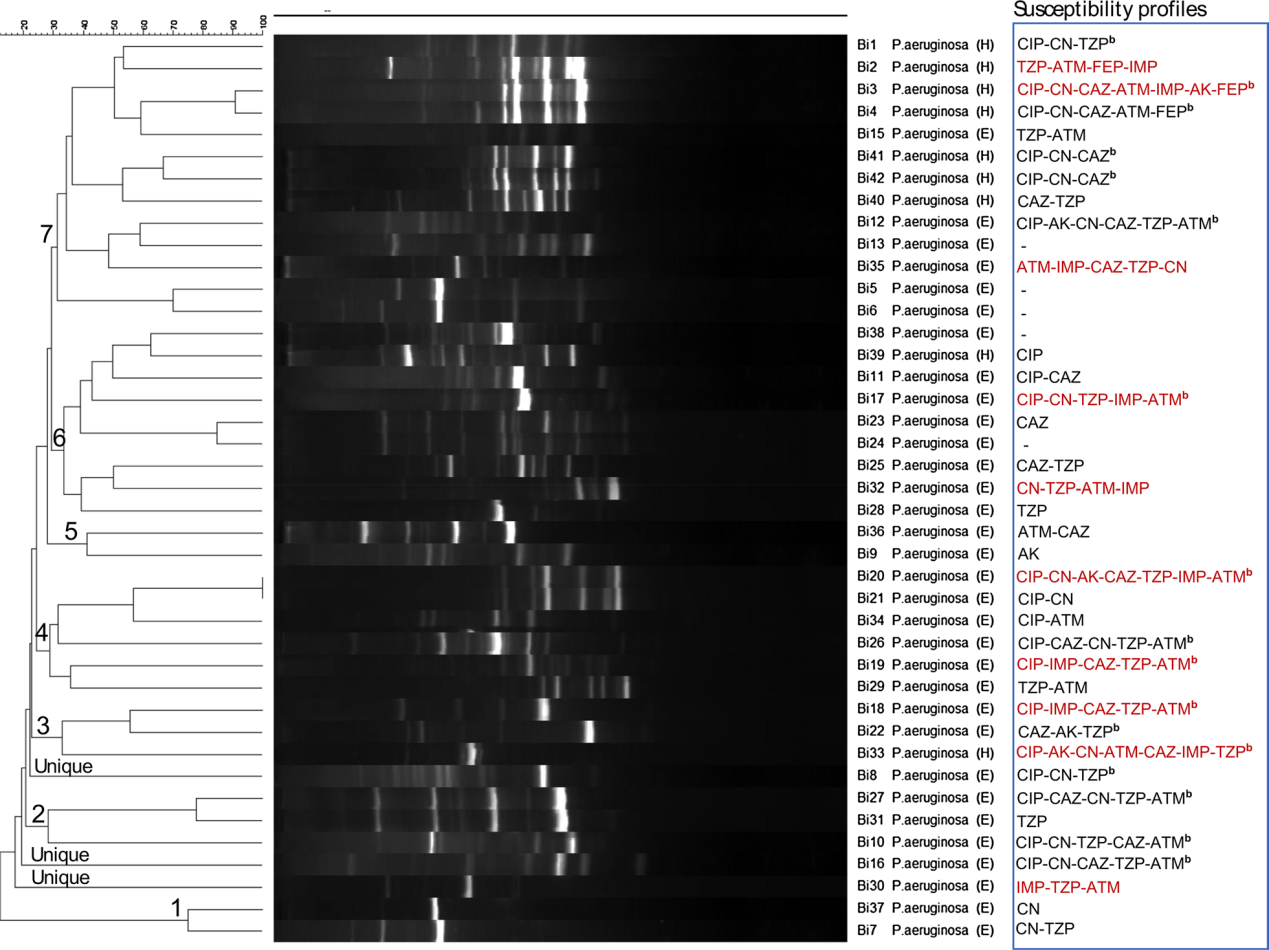
Dendrogram based on Dice’s coefficient of similarity using the UPGMA method implemented by the Bionumerix program showing relationships between *P. aeruginosa* isolates according to BOXAIR-PCR genotyping. *E* isolate from hospital environment; *H* isolate from hospitalized patient. The susceptibility profiles (extreme *right panel*) for carbapenem-resistant isolates are indicated in *brown font*. *AK* Amikacin, *CN* gentamicin, *IMP* imipenem, *MEM* meropenem, *CAZ* ceftazidime, *FEP* cefepime, *ATM* aztreonam, *TZP* piperacillin/tazobactam, *CIP* ciprofloxacin. ^b^Multidrug-resistance pattern

For *P. aeruginosa*, cluster analysis at 100% similarity scale with BOXAIR-PCR, ERIC-PCR, and REP-PCR fingerprints generated one, two and three clusters, respectively, Fig. 3 and Additional file 5: Figures S3, Additional file 6: Figure S4. Isolates 20 and 21 from the environment were considered genetically related as their fingerprints were clustered by both BOXAIR-PCR and REP-PCR analysis, Fig. 3 and Additional file 6: Figure S4. As observed above for Acinetobacter, isolates from a patient and environment (1 and 25, respectively) were also clustered by ERIC-PCR analysis, Additional file 5: Figure S3. Likewise for *P. aeruginosa*, few isolates (10 of 42, 24%) generated fingerprints that were clustered at 100% similarity scale, implying a low level of genetic relatedness but a complex nature of multidrug resistant *P. aeruginosa* clones could be circulating at Mulago Hospital given the diverse antimicrobial susceptibility profiles generated by the clustering isolates, Additional file 2: Table S2.

The Simpson’s index of discrimination (D) for *P. aeruginosa* fingerprints was 0.9829 (BOXAIR-PCR), 0.9756 (ERIC-PCR), and 0.9791 (REP-PCR), indicating that the three genotyping methods successfully discriminated *P. aeruginosa* but BOXAIR-PCR and REP-PCR performed better than ERIC-PCR. For *A. baumannii*, Simpson’s index of discrimination was 0.9632 (BOXAIR-PCR), 0.9608 (ERIC-PCR), and 0.9474 (REP-PCR) indicating that the three methods also successfully discriminated the isolates but BOXAIR-PCR and ERIC-PCR performed better.

## Discussion

In this study, we found the prevalence of multidrug resistant *P. aeruginosa* and *A. baumannii* at Mulago Hospital to be high but comparable to rates reported by Pitout et al. in Kenya [11], Lee et al. in Korea [13] and Gu et al. in China [14]. Additionally, the rates in the current study are lower than those reported by Uma Karthika et al. in India [15]. This could reflect challenges in embracing antibiotic stewardship programmes, or the differences in the amounts of antibiotics consumed in each setting where those studies were conducted [16]. Importantly, in Uganda like many other low-income countries, antibiotics are readily available over the counter in community pharmacies [9, 17] and this portends high rates of antimicrobial resistance in low-income countries.

Furthermore, carbapenem resistance prevalence in this study remains low in isolates from patients in accordance with recent findings at Mulago [8, 10, 18], and significantly lower than rates from Latin America [19]. Yet, carbapenem resistance in this study was high in isolates from the environment, possibly due to the inadequate infection control practices at the hospital [8]. Notably, carbapenem resistant *Acinetobacter* species that were SXT-susceptible were noted. Generally, SXT is not very active against *Acinetobacter* but it was recommended where there are no other options [20], e.g. for infections with pan-resistant strains or in settings where efficacious drugs like carbapenems are expensive [18].

Although the metallo-beta-lactamases were associated with imipenem resistance in this study,  it is important to note that carbapenemases are not the only mechanisms of acquired resistance to carbapenems [21]. Other mechanisms that were not investigated in this study, such as loss or reduced expression of the outer membrane porin OprD, are associated with imipenem resistance; likewise, up-regulation of efflux pumps (e.g. MexAB-OprM) combined with OprD loss is associated with meropenem resistance.

Additionally, it is widely accepted that aztreonam susceptibility combined with resistance to carbapenems and other beta-lactams is a “classic” metallo-beta-lactamase phenotype [21]. However, metallo-beta-lactamase producing strains that are resistant to aztreonam do occur, implying that the “classic” metallo-beta-lactamase pattern is not always seen [21]. Indeed in this study, the prevalence of aztreonam resistance was high among carbapenem resistant isolates. Further, although metallo-beta-lactamase producing strains do not generally hydrolyze monobactams (e.g. aztreonam), metallo-beta-lactamase producing *P. aeruginosa* strains that are resistant to aztreonam have been reported. This is not surprising in that production of a metallo-beta-lactamase does not exclude presence of intrinsic resistance mechanisms in *P. aeruginosa*, e.g. up-regulated efflux pumps or outer membrane impermeability [21] outlined above. Indeed, carbapenem resistant *P. aeruginosa* and *Acinetobacter* strains that are resistant to aztreonam have been reported by researchers worldwide [22]. Taken together, there is surprisingly high level of aztreonam resistance in *P. aeruginosa* isolated from hospital environments [23]. In a study by Santoro et al. [24] on isolates from the hospital wastewater treatment system and clinical specimens in a hospital in Rio de Janeiro city Brazil, 63% of *P. aeruginosa* isolates from sewage exhibited decreased susceptibility to aztreonam while 50% of the clinical isolates were resistant to this drug [24].

In a recent study at Mulago National Referral Hospital, more than three quarters of the Enterobacteriaceae were found to be ESBL-producers [18]. Although resistance to extended-spectrum cephalosporins (aztreonam and ceftazidime) was high in this study, ESBLs and AmpC activity was not detected implying that the genes encoding these enzymes might have not yet spread to *P. aeruginosa* and *Acinetobacter* strains at Mulago. However, we could have missed detecting ESBLs and AmpC producing isolates as the double disc synergy test we used is not very efficient at detecting these enzymes particularly when ESBL activity is low, leading to wide zones of inhibition around cephalosporin/aztreonam discs [25]. As the *AmpC* and *ESBL* genes are transmissible via horizontal gene transfer mechanisms and are associated with multidrug resistance, AmpC and ESBL screening in organisms other than *Escherichia coli*, *Klebsiella pneumoniae*, *Klebsiella oxytoca*, and *Proteus mirabilis* in hospitals where ESBLs and AmpC are encountered is considered an important component of infection control programmes [26]. Therefore, continual surveillance at Mulago for AmpC/ESBL-producing *P. aeruginosa* and *Acinetobacter* species is necessary.

Generally, cluster analysis in this study revealed a low level of genetic relatedness among the isolates. The few clustered isolates point to likely occurrence of complex clones of multidrug resistant *P. aeruginosa* and *A. baumannii* circulating at Mulago Hospital as the isolates exhibited diverse antimicrobial susceptibility patterns. Further studies with a larger number of isolates are necessary to obtain a better picture of the circulating clones at Mulago Hospital. Other researchers who used Rep-PCR genotyping also reported a high level of genetic diversity among *P. aeruginosa* isolated from a hospital in Belo Horizonte, Brazil [22]. Moreover, a nonclonal epidemic population structure of *P. aeruginosa*, supported by lack of evidence for a widespread cystic fibrosis transmissible clone, has been reported [27].

On the other hand, the high genetic diversity among isolates in this study might also point to either a high host diversity from where the bacteria were isolated, or lack of infection control practices. As the only national referral hospital in Uganda, Mulago admits patients from the whole country characterized by variation in geographic landscapes, human populations and human development index (HDI) [9]. Although *P. aeruginosa* and *Acinetobacter* species colonizing hospital settings in high income countries are characterized with clonality, the picture might be different in low-income settings characterized with overcrowding of patients, lack of adequate infection control and proper hygiene. Indeed, hospitals in Uganda are overcrowded with clients from all over the country hence, maintaining environmental hygiene becomes tedious [28]. More to this, the ‘*my five moments for hand* hygiene’ designed for healthcare environments describes recommended levels of bed spacing and occupancy that are normally feasible in high-income countries. Overcrowding of healthcare facilities in low-income countries is often worsened by minimal infection control practices as evidenced by low standards of hand hygiene among health care workers [28]. With low standards of environmental hygiene, bacteria like Pseudomonas are likely to persist in hospital environments where they easily exchange/acquire resistance genetic elements. There is also uncontrolled use of drugs like the third generation cephalosporins in low-income settings and this facilitates selection of diverse drug resistant strains within the healthcare facility [29].

## Conclusions

The prevalence of multidrug resistant *P. aeruginosa* and *A. baumannii* is high at Mulago Hospital. Carbapenem resistance prevalence remains low in isolates from hospitalized patients but high in the environment, possibly due to inadequate infection control practices. As isolates from patients and environment were clustered, hospitalized patients are at high risk of infection with multidrug resistant strains from the environment. Although the repetitive sequence elements on which Rep-PCR genotyping is based are highly conserved throughout the prokaryotic kingdom [30] and have been used extensively in the surveillance of *P. aeruginosa* and *Acinetobacter* species and other bacterial species [31–35], we recommend further studies employing modern genotyping methods, e.g. pulse field gel electrophoresis and/or DNA sequencing-based approaches (the gold standard for bacterial typing) that were not affordable in the current study.

## Methods

### Setting

This was a follow-up surveillance study conducted at Mulago Hospital in Kampala Uganda, following the detection of carbapenem resistant Gram negative bacteria at the hospital in the period 2007–2009 [8, 10]. A total of 736 specimens from hospitalized patients were referred to the Clinical Microbiology and Molecular Diagnostics Laboratories at the College of Health Sciences, Makerere University, for culture and sensitivity testing. Additionally, 100 samples from the hospital environment [water, disinfectants in use (chlorhexidine gluconate), wet sink swabs, wet floors swabs, and swabs of cleaning materials (mops, squeezers)] were randomly collected and similarly processed for isolation of Pseudomonas and Acinetobacter species.

### Identification of *P. aeruginosa* and Acinetobacter species

The sample collection and processing procedure was described in Kateete et al. [8]. Briefly, samples/specimens were processed within 2 h of collection for identification of Gram negative bacteria by following standard microbiological/biochemical procedures [8]. The recovered isolates were first presumptively identified based on colony morphology, Gram-staining properties and standard biochemical characteristics. Characteristic colony morphological features (i.e. colonies with characteristic spreading pattern and serrated edges, fruity sweet-grape smell, and bright green color) were used to identify Pseudomonas. Positive catalase and oxidase tests, negative triple sugar iron and glucose fermentation tests and growth at 42 °C were used to distinguish *P. aeruginosa* from other lactose non-fermenting Gram-negative rods. *Acinetobacter* species were presumptively identified based on negative motility, catalase, oxidase and glucose fermentation tests, and inability to grow under aerobic conditions.

To confirm Acinetobacter isolates to species level, the ‘Phoenix™ Automated Microbiology System’ (Becton and Dickson, Franklin Lakes, NJ, USA) was used; in addition, PCR-amplification followed by DNA sequencing of the species-specific region of the *bla*_OXA-51_ gene intrinsic to *A. baumannii* [5, 36] was performed. To confirm *P. aeruginosa* to species level, and to determine the antimicrobial susceptibility profiles of both *A. baumannii* and *P. aeruginosa* isolates, minimum inhibitory concentrations (MICs) were performed with the ‘Phoenix™ Automated Microbiology System’ according to the manufacturer’s guidelines with minor modifications for Gram negative bacteria as described elsewhere [8, 37]. In addition, the disc diffusion method was performed to determine SXT susceptibility of *A. baumannii* isolates, according to the Clinical and Laboratory Standards Institute (CLSI) guidelines. Quality control and maintenance for the ‘Phoenix™ Automated Microbiology System’ were performed as recommended by the manufacturer using reference strains of *E. coli* ATCC 25922 and *P. aeruginosa* ATCC 27853. Phoenix™ default MIC-breakpoints were described in Kateete et al. [8].

### Metallo-β-lactamase assays

All the carbapenem-resistant isolates were tested for metallo-β-lactamase activity as bacteria producing these enzymes are characterized by rapid spread worldwide [5]. Assays were performed with the imipenem-EDTA (Ethylenediaminetetraacetic acid) double-disk synergy test using *K. pneumoniae* ATCC 700603 and *E. coli* ATCC 25922 as indicator strains [10, 38, 39]. An overnight liquid culture of the test isolate was adjusted to a turbidity of 0.5 McFarland standard and spread on the surface of a Mueller–Hinton Agar (MHA) plate. Two imipenem discs (10 μg each) were placed on the agar 15 mm apart (center-to-center); 10 μl of 0.5 M EDTA was added to one of the imipenem disc to get a desired concentration of 750 μg. After incubating at 37 °C overnight, the difference in the inhibition zone diameter that was ≥5 mm between the imipenem disc supplemented with EDTA and imipenem disc alone was interpreted as positive for metallo-β-lactamase production [21, 39]. *K. pneumoniae* strains ATCC BAA-1705 and ATCC BAA-1706 served as positive and negative controls, respectively; strain BAA-1705 possesses a *K. pneumoniae* carbapenemase KPC-2 that is highly active against cephamycins, carbapenems, and to a substrate range of ESBLs [40]. Positive or negative results were interpreted according to the CLSI guidelines and the UK Standards for Microbiology Investigations [21, 39, 41].

### ESBL and AmpC screening

Isolates that exhibited resistance to extended-spectrum cephalosporins (ceftazidime and aztreonam) were subjected to the double disk synergy test to detect production of ESBLs as described elsewhere [26], by placing ceftazidime, cefotaxime, aztreonam and ceftriaxone discs (30 µg each) at 20 mm distance (center-to-center) from amoxicillin (20 µg) and clavulanic acid (10 µg) discs. ESBL activity was inferred if the cephalosporin zone was expanded by the clavulanic acid containing disk. Ceftazidime and/or aztreonam resistant isolates were also tested for production of plasmid-mediated AmpC as previously described [26] using MHA plates and ceftazidime (30 µg) and cefoxitin (30 µg) discs placed 20 mm (center-to-center). AmpC-production was considered when isolates showed blunting of ceftazidime zone of inhibition adjacent to cefoxitin disc.

### Genotyping, electrophoresis and analysis of DNA fingerprints

Genotyping was performed with Rep-PCR assays (REP-, BOXAIR- and ERIC-PCRs). Initially, we followed the procedure described by Syrmis et al. [35] to optimize the concentration of Mg++, template DNA, Taq polymerase and PCR primers, using four unrelated isolates of *P. aeruginosa* and *A. baumannii*. Chromosomal DNA used as templates in PCRs was extracted by the Cetyltrimethyl Ammonium Bromide (CTAB) method [42, 43] and dissolved in 100 μl of sterile Tris–EDTA (TE) buffer. 100 ng of the extracted DNA was used as template in the PCRs. REP-, BOXAIR- and ERIC-PCRs were performed using previously described primers and conditions [32, 44], with minor modifications to suit our setting. Ten microliters each of the PCR products was size-fractionated by agarose gel electrophoresis (1.5% w/v agarose) at constant voltage of 40 V in 1× TAE buffer (Tris–acetate pH 8.3, 40 mM Tris, 20 mM acetic acid, 1 mM EDTA) for 8 h in a horizontal electrophoretic apparatus (Bio-Rad Inc.). For quality control and uniformity in DNA migration during electrophoresis, all the gels were electrophoresed for equal amount of time. DNA size markers contained a mixture of the 50 base pairs (bp) DNA ladder (50–1350 bp size range) plus λ DNA-*Hind*III digest DNA ladder (2027–23,130 bp visible size range) (New England Biolabs Inc.). REP-, ERIC-, and BOXAIR-PCR profiles were visualized under ultraviolet (UV) light after staining the gels with ethidium bromide for 30 min. Images were captured and stored as TIFF files using BioDoc-it Imaging system (UVP, Cambridge, UK).

DNA fingerprints were analyzed with the BioNumerics software version 4 (Applied Maths NV, Sint-Martens-Latem, Belgium) as described elsewhere [34, 35, 45, 46]. The similarity-analysis of the results of each genotyping assay was calculated using Dice coefficient; cluster analysis of the similarity matrices was generated using Unweighted Pair Group Method with Arithmetic Mean (UPGMA) algorithm. The relationship between profiles was expressed as dendrograms; only visible bands were used to construct the similarity matrices and dendrograms. Variation in band intensity was not considered as genetic difference. Isolates were considered identical when all the scored bands in each pattern had similar migration patterns/distance. As few isolates shared similar migration patterns, the criterion for relatedness was taken as profiles with ≥85% similar migration patterns/distance [35]. The Simpson’s index of diversity [47, 48] was used to estimate discrimination indices (D values) for the three typing methods.

### Quality control

Controls for Rep-PCR genotyping included known well characterized strains *K. pneumoniae* DSMZ 9377, *E. coli* ATCC 25922 and *P. aeruginosa* ATCC 27853. We optimized Rep-PCR assays using the procedure described for Yersinia [46] and adapted it to *P. aeruginosa* and *A. baumannii*. Genotyping was performed at least twice in separate assays to ensure reproducibility of the patterns; BOXAIR-PCR, ERIC-PCR, and REP-PCRs were repeated for five unrelated isolates of *P. aeruginosa* and *A. baumannii*. PCRs and electrophoresis were performed in two separate trials starting from the same DNA preparation, using the same PCR reagents. None of the isolates showed qualitative differences in the fingerprints. We also investigated the effect of the DNA preparation method using chromosomal DNA extracted with two in-house methods of CTAB and boiling; although CTAB-prepared DNA consistently yielded fingerprints with stronger band intensities, no significant difference in band sizes was detected suggesting stability of our Rep-PCR fingerprints.

## Additional files

**Additional file 1: Table S1.** Antimicrobial resistance profiles of *P. aeruginosa* and *A. baumannii.*

**Additional file 2: Table S2.** Antimicrobial resistance profiles of isolates that were clustered at 100% similarity scale. The diverse antimicrobial susceptibility profiles exhibited by the clustered isolates reveal a complex nature of multidrug resistant *P. aeruginosa* and *A. baumannii* clones circulating at Mulago Hospital.

**Additional file 3: Figure S1.** Representative image showing agarose gel electrophoresis of *P. aeruginosa* and *A. baumannii* PCR fingerprints following BOXAIR-PCR genotyping. Panels A and B, lanes 18-42, *P. aeruginosa* fingerprints; panel B, lanes 43-51, *A. baumannii* fingerprints; lane M, DNA size marker that was reconstituted by mixing 50 base pair-DNA ladder (50-1350 bp size range) with λ DNA-*Hind*III digest-DNA ladder (2027-23,130 bp visible size range). Carbapenem-resistant isolates are indicated in red font.

**Additional file 4: Figure S2.** Dendrogram based on Dice’s coefficient of similarity using the UPGMA method implemented by the Bionumerix program showing relationships between *A. baumannii* isolates according to ERIC-PCR genotyping. (E), isolate from hospital environment; (H), isolate from hospitalized patient. The susceptibility profiles (extreme right panel) for carbapenem-resistant isolates are indicated in brown font; AK, Amikacin; CN, Gentamicin; IMP, Imipenem; MEM, Meropenem; CAZ, Ceftazidime; FEP, Cefepime; ATM, Aztreonam; TZP, Piperacillin/tazobactam; CIP, Ciprofloxacin; SXT, Trimethoprim/sulfamethoxazole. ^b^Multidrug-resistance pattern; ^c^SXT-susceptible carbapenem-resistant *A. baumannii*.

**Additional file 5: Figure S3.** Dendrogram based on Dice’s coefficient of similarity using the UPGMA method implemented by the Bionumerix program showing relationships between *P. aeruginosa* isolates according to ERIC-PCR genotyping. (E), isolate from hospital environment; (H), isolate from hospitalized patient. The susceptibility profiles (extreme right panel) for carbapenem-resistant isolates are indicated in brown font; AK, Amikacin; CN, Gentamicin; IMP, Imipenem; MEM, Meropenem; CAZ, Ceftazidime; FEP, Cefepime; ATM, Aztreonam; TZP, Piperacillin/tazobactam; CIP, Ciprofloxacin. ^b^Multidrug-resistance pattern.

**Additional file 6: Figure S4.** Dendrogram based on Dice’s coefficient of similarity using the UPGMA method implemented by the Bionumerix program showing relationships between *P. aeruginosa* isolates according to REP-PCR genotyping. (E), isolate from hospital environment; (H), isolate from hospitalized patient. The susceptibility profiles (extreme right panel) for carbapenem-resistant isolates are indicated in brown font; AK, Amikacin; CN, Gentamicin; IMP, Imipenem; MEM, Meropenem; CAZ, Ceftazidime; FEP, Cefepime; ATM, Aztreonam; TZP, Piperacillin/tazobactam; CIP, Ciprofloxacin. ^b^Multidrug-resistance pattern.

## Abbreviations

ICU: intensive care unit
MICs: minimum inhibitory concentrations
SXT: trimethoprim/sulfamethoxazole
CLSI: Clinical and Laboratory Standards Institute
EDTA: ethylenediaminetetraacetic acid
KPC: *Klebsiella pneumoniae* carbapenemase
ESBLs: extended spectrum beta-lactamases
Rep-PCR: repetitive elements-based PCR
TE: Tris–EDTA
TAE: tris acetic acid EDTA buffer
UPGMA: Unweighted Pair Group Method with Arithmetic Mean
UV: ultraviolet
