# Supplementary material for: Genotypic diversity among multidrug resistant Pseudomonas aeruginosa and Acinetobacter species at Mulago Hospital in Kampala, Uganda
Source: BMC Res Notes. 2017 Jul 14;10:284. doi: 10.1186/s13104-017-2612-y (PMC5513047; doi:10.1186/s13104-017-2612-y)
Supplement: Supplementary file 1 — Additional file 1: Table S1. Antimicrobial resistance profiles of P. aeruginosa and A. baumannii. [file 13104_2017_2612_MOESM1_ESM.docx]

**Table S1:** Antimicrobial resistance profiles of *P. aeruginosa* and *A. baumannii*

| **Lab No.** | **Isolate** | **Species** | **Source** | **Resistance pattern** | **IMP/**  **EDTA^a^** | **ESBL / AmpC** |
| --- | --- | --- | --- | --- | --- | --- |
| 1 | 2497 | *Pseudomonas aeruginosa* | Hospitalized patient | CIP-CN-TZP**^b^** |  | NA |
| 2 | 1665 | *Pseudomonas aeruginosa* | Hospitalized patient | **TZP-ATM-FEP-IMP** | + | - |
| 3 | 0327 | *Pseudomonas aeruginosa* | Hospitalized patient | **CIP-CN-CAZ-ATM-IMP-AK-FEP^b^** | + | - |
| 4 | 1246 | *Pseudomonas aeruginosa* | Hospitalized patient | CIP-CN-CAZ-ATM-FEP**^b^** | NA | - |
| 5 | J001 | *Pseudomonas aeruginosa* | Environment | - | NA | NA |
| 6 | J004 | *Pseudomonas aeruginosa* | Environment | - | NA | NA |
| 7 | J008 | *Pseudomonas aeruginosa* | Environment | CN-TZP | NA | NA |
| 8 | J009-2 | *Pseudomonas aeruginosa* | Environment | CIP-CN-TZP**^b^** | NA | NA |
| 9 | J010-2 | *Pseudomonas aeruginosa* | Environment | AK | NA | NA |
| 10 | J018-3 | *Pseudomonas aeruginosa* | Environment | CIP-CN-TZP-CAZ-ATM**^b^** | NA | - |
| 11 | J020-1 | *Pseudomonas aeruginosa* | Environment | CIP-CAZ | NA | - |
| 12 | J041-2 | *Pseudomonas aeruginosa* | Environment | CIP-AK-CN-CAZ-TZP-ATM**^b^** | NA | - |
| 13 | J044-1 | *Pseudomonas aeruginosa* | Environment | - | NA | NA |
| 14 | J045-1 | *Pseudomonas aeruginosa* | Environment | TZP | NA | NA |
| 15 | J046-1 | *Pseudomonas aeruginosa* | Environment | TZP-ATM | NA | - |
| 16 | J047-2 | *Pseudomonas aeruginosa* | Environment | CIP-CN-CAZ-TZP-ATM**^b^** | NA | - |
| 17 | J049-1 | *Pseudomonas aeruginosa* | Environment | **CIP-CN-TZP-IMP-ATM**^b^ | + | - |
| 18 | J052-1 | *Pseudomonas aeruginosa* | Environment | **CIP-IMP-CAZ-TZP-ATM**^b^ | + | - |
| 19 | J053-2 | *Pseudomonas aeruginosa* | Environment | **CIP-IMP-CAZ-TZP-ATM**^b^ | - | - |
| 20 | J059 | *Pseudomonas aeruginosa* | Environment | **CIP-CN-AK-CAZ-TZP-IMP-ATM^b^** | - | - |
| 21 | J061 | *Pseudomonas aeruginosa* | Environment | CIP-CN | NA | NA |
| 22 | J063-2 | *Pseudomonas aeruginosa* | Environment | CAZ-AK-TZP**^b^** | NA | - |
| 23 | J064-1 | *Pseudomonas aeruginosa* | Environment | CAZ | NA | - |
| 24 | J064-2 | *Pseudomonas aeruginosa* | Environment | - | NA | NA |
| 25 | J071-1 | *Pseudomonas aeruginosa* | Environment | CAZ-TZP | NA | - |
| 26 | J078-1 | *Pseudomonas aeruginosa* | Environment | CIP-CAZ-CN-TZP-ATM**^b^** | NA | - |
| 27 | J079-1 | *Pseudomonas aeruginosa* | Environment | CIP-CAZ-CN-TZP-ATM**^b^** | NA | - |
| 28 | J079-2 | *Pseudomonas aeruginosa* | Environment | TZP | NA | NA |
| 29 | J079-3 | *Pseudomonas aeruginosa* | Environment | TZP-ATM | NA | - |
| 30 | J081-1 | *Pseudomonas aeruginosa* | Environment | **IMP-TZP-ATM** | + | - |
| 31 | J081-2 | *Pseudomonas aeruginosa* | Environment | TZP | NA | NA |
| 32 | J081-3 | *Pseudomonas aeruginosa* | Environment | **CN-TZP-ATM-IMP** | - | - |
| 33 | J085 | *Pseudomonas aeruginosa* | Hospitalized patient | **CIP-AK-CN-ATM-CAZ-IMP-TZP^b^** |  | - |
| 34 | J086-1 | *Pseudomonas aeruginosa* | Environment | CIP-ATM | NA | - |
| 35 | J096 | *Pseudomonas aeruginosa* | Environment | **ATM-IMP-CAZ-TZP-CN** | - | - |
| 36 | J099-1 | *Pseudomonas aeruginosa* | Environment | ATM-CAZ | NA | - |
| 37 | J100-2 | *Pseudomonas aeruginosa* | Environment | CN | NA | NA |
| 38 | J101-2 | *Pseudomonas aeruginosa* | Environment | - | NA | NA |
| 39 | J104-4 | *Pseudomonas aeruginosa* | Hospitalized patient | CIP | NA | NA |
| 40 | J105 | *Pseudomonas aeruginosa* | Hospitalized patient | CAZ-TZP | NA | - |
| 41 | J106 | *Pseudomonas aeruginosa* | Hospitalized patient | CIP-TZP | NA | NA |
| 42 | 1327 | *Pseudomonas aeruginosa* | Hospitalized patient | CIP-CN-CAZ**^b^** | NA | - |
| 43 | Code5 | *Acinetobacter baumannii* | Hospitalized patient | **CIP-TZP-IMP-MEM^c^** | + | NA |
| 44 | J005 | *Acinetobacter baumannii* | Environment | CIP-TZP-SXT-ATM**^b^** | NA | - |
| 45 | J008 | *Acinetobacter baumannii* | Environment | SXT-CN | NA | NA |
| 46 | J028-2 | *Acinetobacter baumannii* | Environment | **CN-TZP-IMP-ATM^c^** | + | - |
| 47 | J031 | *Acinetobacter baumannii* | Environment | TZP-SXT | NA | NA |
| 48 | J041-1 | *Acinetobacter baumannii* | Environment | - | NA | NA |
| 49 | J044-2 | *Acinetobacter baumannii* | Environment | **TZP-IMP-ATM^c^** | + | - |
| 50 | J046-2 | *Acinetobacter baumannii* | Environment | **SXT-TZP-IMP** | - | NA |
| 51 | J052-2 | *Acinetobacter baumannii* | Environment | **CIP-SXT-TZP-IMP-ATM^b^** | + | - |
| 52 | J054 | *Acinetobacter baumannii* | Environment | **CIP-SXT-CAZ-TZP-ATM-IMP^b^** | + | - |
| 53 | J060 | *Acinetobacter baumannii* | Environment | TZP-SXT | NA | NA |
| 54 | J087-1 | *Acinetobacter baumannii* | Environment | TZP-SXT | NA | NA |
| 55 | J093 | *Acinetobacter baumannii* | Environment | **IMP-TZP^c^** | + | NA |
| 56 | J094-1 | *Acinetobacter baumannii* | Environment | TZP | NA | NA |
| 57 | R103 | *Acinetobacter baumannii* | Hospitalized patient | CIP-CN-AK-ATM-TZP**^b^** | NA | - |
| 58 | 194 | *Acinetobacter baumannii* | Hospitalized patient | CIP-SXT-CAZ**^b^** | NA | - |
| 59 | 207 | *Acinetobacter baumannii* | Hospitalized patient | CIP-CN-TZP-CAZ-FEP-AK-ATM-SXT**^b^** | NA | - |
| 60 | 182 | *Acinetobacter baumannii* | Hospitalized patient | CIP-CN-AK-SXT-CAZ-ATM**^b^** | NA | - |
| 61 | 179 | *Acinetobacter baumannii* | Hospitalized patient | CN-CAZ-CIP- SXT**^b^** | NA | - |
| 62 | R105 | *Acinetobacter baumannii* | Hospitalized patient | CN-CAZ | NA | - |

Pan-susceptible *P. aeruginosa,* 12% (5/42)

- Hospitalized patients: 0 (none of the nine isolates from patients was pan-susceptible)
- Environment: 16% (5/32)

Pan-susceptible *A. baumannii*, 5% (1/20)

- Hospitalized patients: 0 (none of the seven isolates from patients was pan-susceptible)
- Environment: 8% (1/13)

Carbapenem resistant *P. aeruginosa* 24%, (10/42)

- Hospitalized patients: 0.4% (3/736), in isolates 33% (3/9)
- Environment: 22% (7/32)

Carbapenem resistant *A. baumannii*, 35%, (7/20)

- Hospitalized patients: 0.1% (1/736), in isolates 14%, (1/7)
- Environment: 46% (6/13)

Multidrug-resistance

- *P. aeruginosa* 38% (16/42)
- *A. baumannii*, 40% (8/20)

NA, Not applicable; +, Positive; -, Negative; ^a^Metallo-β-lactamase activity with imipenem-EDTA test; **^b^**Multidrug-resistance pattern. In bold face font are profiles with carbapenem-resistance. **^c^**SXT-susceptible carbapenem-resistant *A. baumannii*.

AK, Amikacin; CN, Gentamicin; IMP, Imipenem; MEM, Meropenem; CAZ, Ceftazidime; FEP, Cefepime; ATM, Aztreonam; TZP, Piperacillin/tazobactam; CIP, Ciprofloxacin; SXT, Trimethoprim/sulfamethoxazole
