# Supplementary material for: Genotypic diversity among multidrug resistant Pseudomonas aeruginosa and Acinetobacter species at Mulago Hospital in Kampala, Uganda
Source: BMC Res Notes. 2017 Jul 14;10:284. doi: 10.1186/s13104-017-2612-y (PMC5513047; doi:10.1186/s13104-017-2612-y)
Supplement: Supplementary file 2 — Additional file 2: Table S2. Antimicrobial resistance profiles of isolates that were clustered at 100% similarity scale. The diverse antimicrobial susceptibility profiles exhibited by the clustered isolates reveal a complex nature of multidrug resistant P. aeruginosa and A. baumannii clones circulating at Mulago Hospital. [file 13104_2017_2612_MOESM2_ESM.docx]

**Table S2:** Antimicrobial resistance profiles of isolates clustered at 100% similarity scale.

| **Cluster No.** | **Isolate^a^** | **Species** | **Drug resistance pattern^b^** | **Rep-PCR method** |
| --- | --- | --- | --- | --- |
| 1 | 207 (ER59) | *Acinetobacter baumannii* | CIP-CN-CAZ-AK-SXT-FEP-ATM |  |
|  | R105 (ER62) | *Acinetobacter baumannii* | CN-CAZ | ERIC-PCR |
| 2 | J060 (ER53) | *Acinetobacter baumannii* | CN-TZP-SXT |  |
|  | J094-1 (ER56) | *Acinetobacter baumannii* | TZP |  |
|  | 207 (RRep59) | *Acinetobacter baumannii* | CIP-CN-SXT-CAZ-ATM-AK-FEP |  |
| 3 | 182 (RRep60) | *Acinetobacter baumannii* | CIP-CN-SXT-CAZ-ATM-TZP | REP-PCR |
|  | R105 (RRep62) | *Acinetobacter baumannii* | CN-CAZ |  |
| 1 | J059 (Bi20) | *Pseudomonas aeruginosa* | CIP-CN-AK-CAZ-TZP-ATM-IMP^R^ | BOXAIR-PCR |
|  | J061 (Bi21) | *Pseudomonas aeruginosa* | CIP-CN |  |
| 2 | J008 (ER7) | *Pseudomonas aeruginosa* | CN-TZP |  |
|  | J045-1 (ER14) | *Pseudomonas aeruginosa* | TZP | ERIC-PCR |
| 3 | 2497 (ER1) | *Pseudomonas aeruginosa* | CIP-CN-TZP |  |
|  | J071-1 (ER25) | *Pseudomonas aeruginosa* | CAZ-TZP |  |
| 4 | 2497 (RRep1) | *Pseudomonas aeruginosa* | CIP-CN-TZP |  |
|  | 0327 (RRep3) | *Pseudomonas aeruginosa* | CIP-CN-TZP-AK-CAZ-ATM-MEM-FEP-IMP^R^ |  |
| 5 | J059 (RRep20) | *Pseudomonas aeruginosa* | CIP-CN-AK-CAZ-TZP-ATM-IMP^R^ |  |
|  | J061 (RRep21) | *Pseudomonas aeruginosa* | CIP-CN | REP-PCR |
|  | J105 (RRep40) | *Pseudomonas aeruginosa* | CAZ-TZP |  |
| 6 | J106 (RRep41) | *Pseudomonas aeruginosa* | TZP |  |
|  | 1327 (RRep42) | *Pseudomonas aeruginosa* | CIP-CN-CAZ-TZP |  |

AK, Amikacin; CN, Gentamicin; IMP, Imipenem; MEM, Meropenem; CAZ, Ceftazidime; FEP, Cefepime; ATM, Aztreonam; TZP, Piperacillin/tazobactam; CIP, Ciprofloxacin; SXT, Trimethoprim/sulfamethoxazole

**^a^**In parenthesis are code numbers used in cluster analysis.

**^b^**The diverse antimicrobial susceptibity profiles exhibited by the clustered isolates reveal a complex nature of multidrug resistant *P. aeruginosa* and *A. baumannii* clones circulating at Mulago Hospital. Resistance patterns common to all isolates in a cluster are highlighted.

**^R^**Only three carbapenem-resistant isolates were clustered at 100% similarity scale.
