# Supplementary figures and images for: Genotypic diversity among multidrug resistant Pseudomonas aeruginosa and Acinetobacter species at Mulago Hospital in Kampala, Uganda
Source: BMC Res Notes. 2017 Jul 14;10:284. doi: 10.1186/s13104-017-2612-y (PMC5513047; doi:10.1186/s13104-017-2612-y)

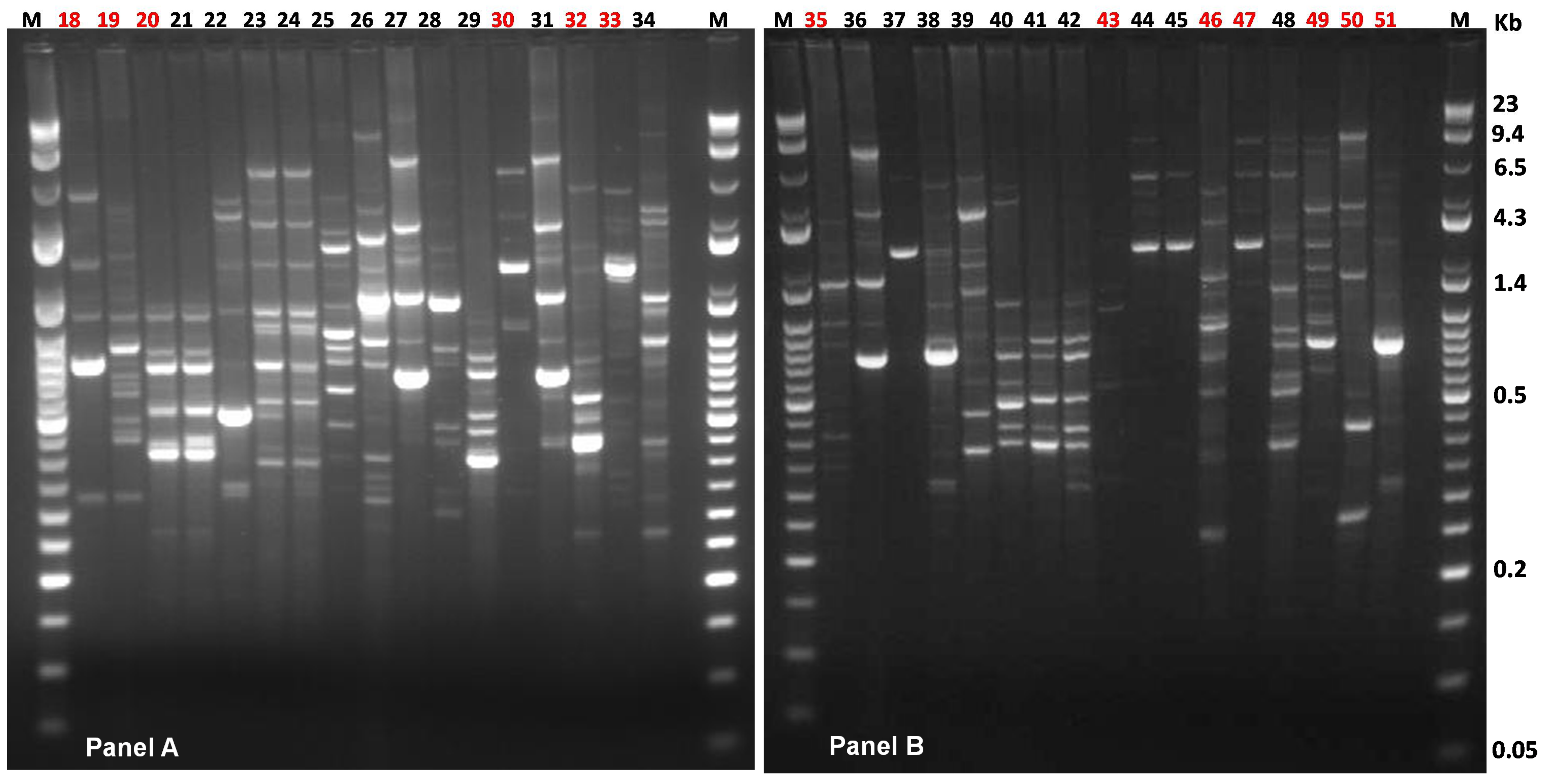

Supplement: Supplementary file 3 — Additional file 3: Figure S1. Representative image showing agarose gel electrophoresis of P. aeruginosa and A. baumannii PCR fingerprints following BOXAIR-PCR genotyping. Panels A and B, lanes 18-42, P. aeruginosa fingerprints; panel B, lanes 43-51, A. baumannii fingerprints; lane M, DNA size marker that was reconstituted by mixing 50 base pair-DNA ladder (50-1350 bp size range) with λ DNA-HindIII digest-DNA ladder (2027-23,130 bp visible size range). Carbapenem-resistant isolates are indicated in red font. [file 13104_2017_2612_MOESM3_ESM.tiff]

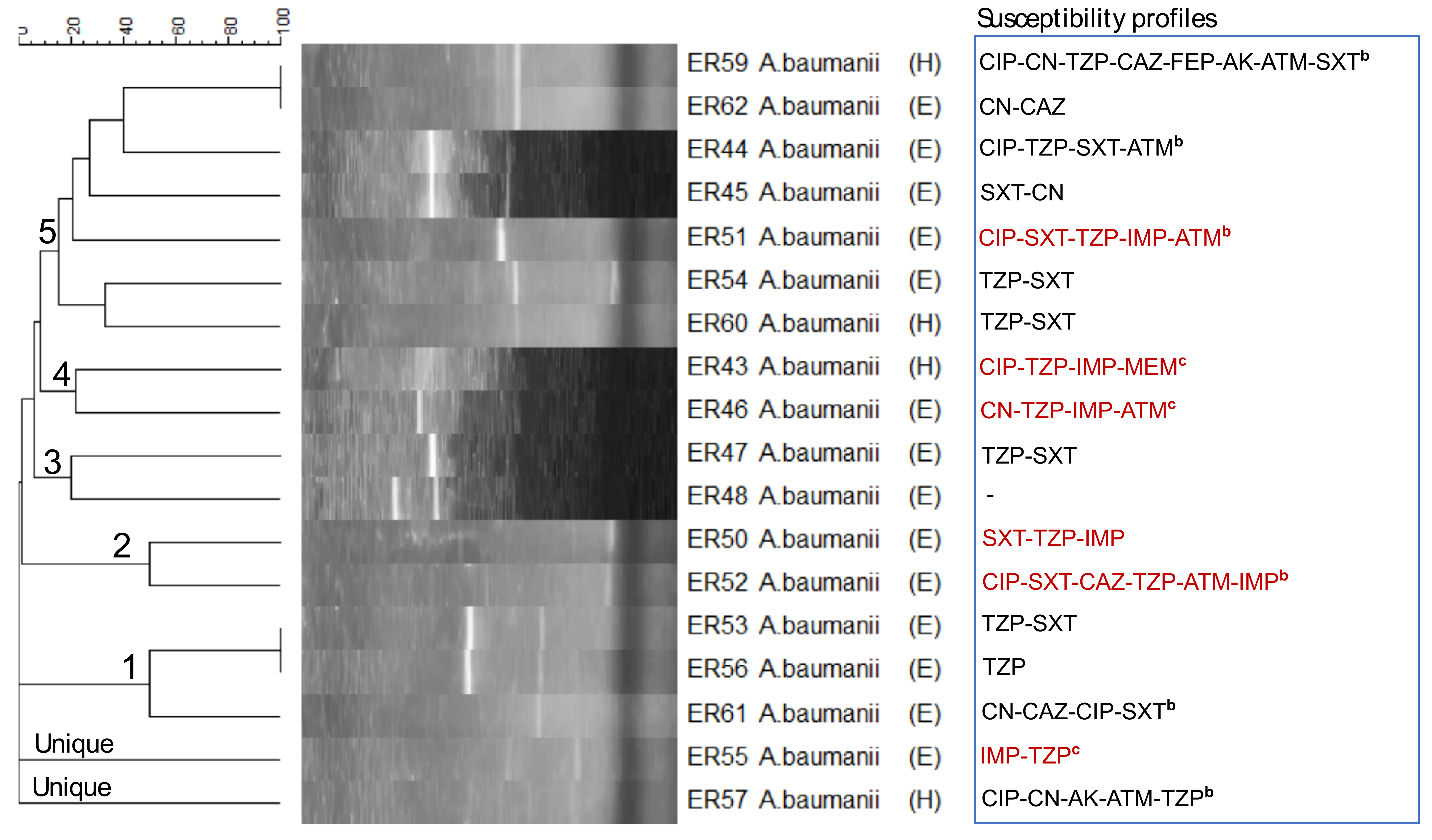

Supplement: Supplementary file 4 — Additional file 4: Figure S2. Dendrogram based on Dice’s coefficient of similarity using the UPGMA method implemented by the Bionumerix program showing relationships between A. baumannii isolates according to ERIC-PCR genotyping. (E), isolate from hospital environment; (H), isolate from hospitalized patient. The susceptibility profiles (extreme right panel) for carbapenem-resistant isolates are indicated in brown font; AK, Amikacin; CN, Gentamicin; IMP, Imipenem; MEM, Meropenem; CAZ, Ceftazidime; FEP, Cefepime; ATM, Aztreonam; TZP, Piperacillin/tazobactam; CIP, Ciprofloxacin; SXT, Trimethoprim/sulfamethoxazole. bMultidrug-resistance pattern; cSXT-susceptible carbapenem-resistant A. baumannii. [file 13104_2017_2612_MOESM4_ESM.tiff]

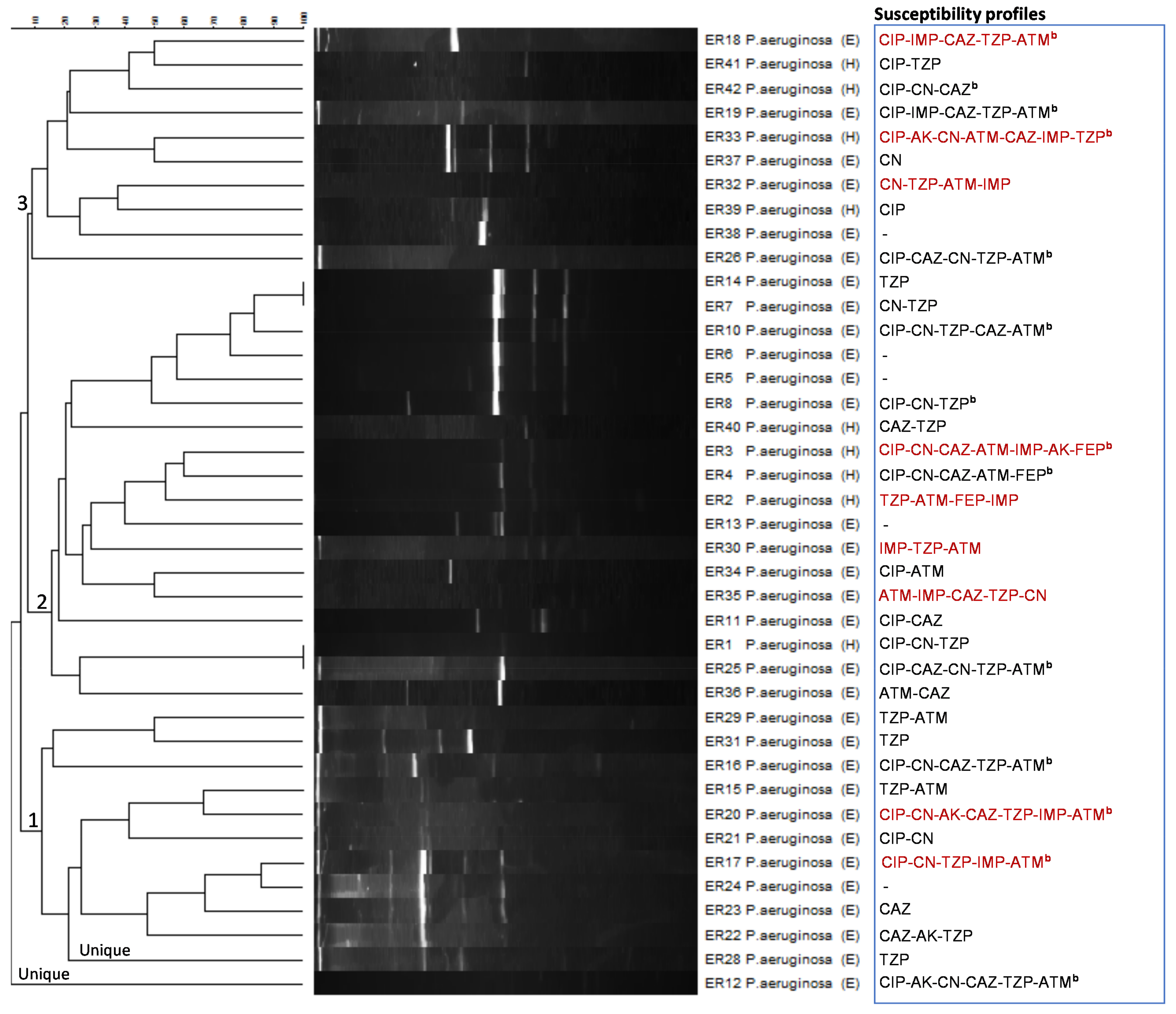

Supplement: Supplementary file 5 — Additional file 5: Figure S3. Dendrogram based on Dice’s coefficient of similarity using the UPGMA method implemented by the Bionumerix program showing relationships between P. aeruginosa isolates according to ERIC-PCR genotyping. (E), isolate from hospital environment; (H), isolate from hospitalized patient. The susceptibility profiles (extreme right panel) for carbapenem-resistant isolates are indicated in brown font; AK, Amikacin; CN, Gentamicin; IMP, Imipenem; MEM, Meropenem; CAZ, Ceftazidime; FEP, Cefepime; ATM, Aztreonam; TZP, Piperacillin/tazobactam; CIP, Ciprofloxacin. bMultidrug-resistance pattern. [file 13104_2017_2612_MOESM5_ESM.tiff]

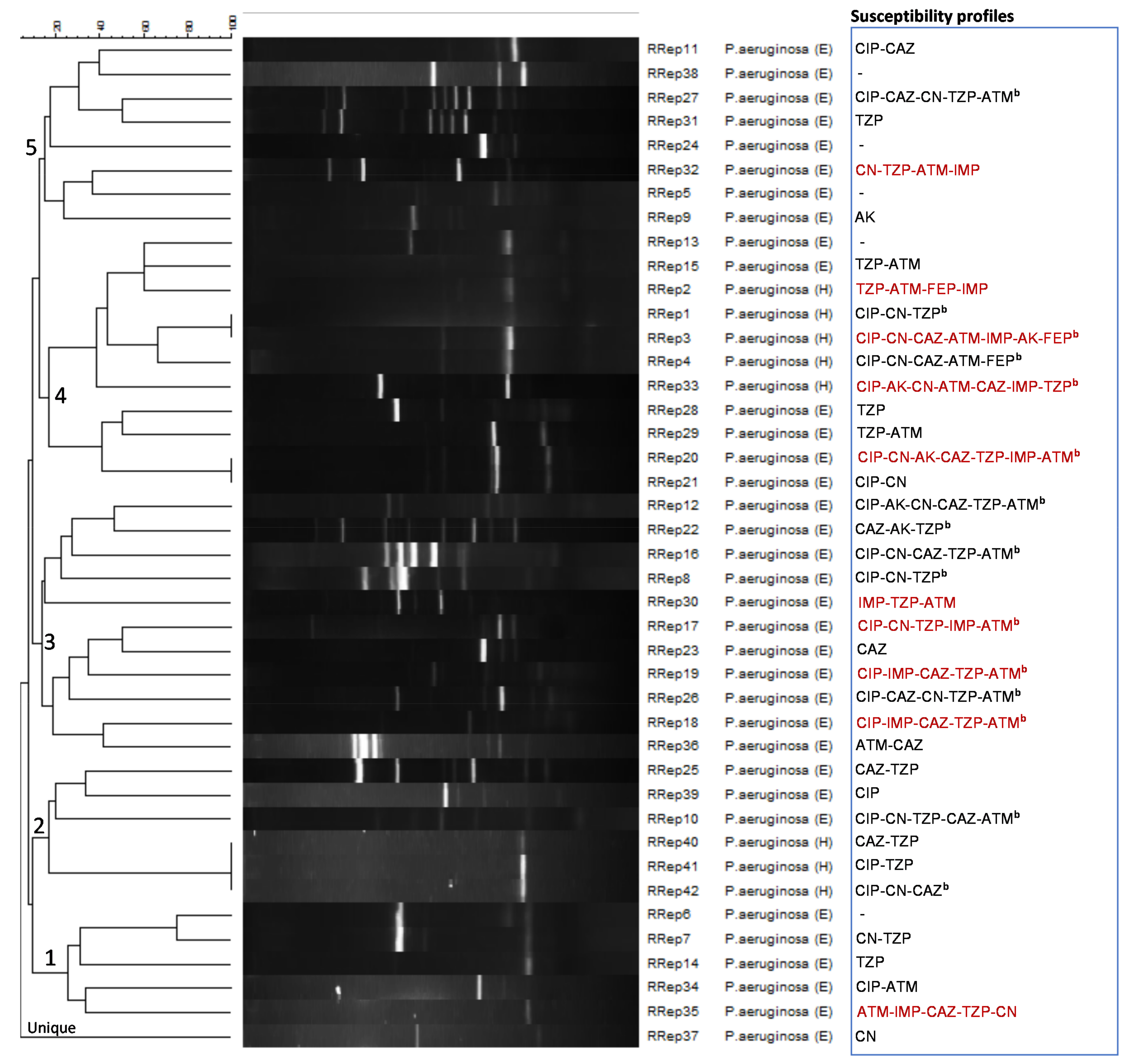

Supplement: Supplementary file 6 — Additional file 6: Figure S4. Dendrogram based on Dice’s coefficient of similarity using the UPGMA method implemented by the Bionumerix program showing relationships between P. aeruginosa isolates according to REP-PCR genotyping. (E), isolate from hospital environment; (H), isolate from hospitalized patient. The susceptibility profiles (extreme right panel) for carbapenem-resistant isolates are indicated in brown font; AK, Amikacin; CN, Gentamicin; IMP, Imipenem; MEM, Meropenem; CAZ, Ceftazidime; FEP, Cefepime; ATM, Aztreonam; TZP, Piperacillin/tazobactam; CIP, Ciprofloxacin. bMultidrug-resistance pattern. [file 13104_2017_2612_MOESM6_ESM.tiff]
